# Utilisation of hormone replacement therapy in Arab countries: a systematic review

**DOI:** 10.3389/fgwh.2026.1722268

**Published:** 2026-02-09

**Authors:** Nasrin Al Zadjali, Celine Tabche, Zeenah Atwan, Salman Rawaf

**Affiliations:** 1 Department of Primary Health Care, Muscat Governorate, Directorate General of Health Services, Ministry of Health, Muscat, Oman; 2 WHO Collaborating Centre for Public Health Education and Training, School of Public Health, Imperial College London, London, United Kingdom; 3 Department of Microbiology, Central Laboratory, College of Medicine, University of Basrah, Basrah, Iraq

**Keywords:** Arab countries, hormone replacement therapy, menopausal hormone therapy, menopause, public health, women's health

## Abstract

**Background:**

Menopause is a major transition in women's health and may be associated with vasomotor symptoms and increased risk of osteoporosis and cardiovascular disease. Hormone replacement therapy (HRT) is an effective treatment option, yet uptake is reported to be limited in many settings. This systematic review aimed to synthesise evidence on factors associated with HRT uptake and utilisation in Arab countries and to summarise proposed strategies to support informed decision-making.

**Methods:**

We searched Embase, MEDLINE, WHO IRIS, Cochrane Library, PubMed, and Scopus for studies published up to March 2025. Eligible studies examined HRT uptake/utilisation, knowledge, perceptions, or attitudes among women in the 22 Arab countries. Two reviewers screened records using Covidence, with disagreements resolved by a third reviewer. Study quality was assessed using the Newcastle–Ottawa Scale adapted for cross-sectional studies. Findings were synthesised thematically. PROSPERO: CRD420251007430.

**Findings:**

Fifteen cross-sectional studies met inclusion criteria. HRT uptake was generally low; in 11 studies fewer than 20% of participants reported use, with reported prevalence ranging from 0% to 48% across settings. Factors positively associated with uptake included higher educational attainment, employment, healthcare provider influence and access to consultation, and greater baseline knowledge of menopause and HRT. Barriers included risk concerns, cultural conservatism, and preference for herbal or “natural” remedies. In several studies, media was a primary source of menopause-related information, while physician-led counselling was less frequently reported.

**Conclusion:**

Evidence from Arab countries indicates predominantly low HRT utilisation, shaped by knowledge gaps, risk perceptions, and socio-cultural factors, alongside variable healthcare-provider engagement. Studies most commonly recommended community education and strengthened clinician communication to support informed decision-making.

**Systematic Review Registration:**

https://www.crd.york.ac.uk/PROSPERO/view/CRD420251007430, PROSPERO CRD420251007430.

## 1 Introduction

Menopause is a normal transition phase in women's lives; however, what is not well known is that 85% of women experience one or more distressing symptoms due to hormonal imbalance (1, 2). The World Health Organization (WHO) defines menopause as having occurred after 12 consecutive months without a menstrual period at a mean age of 51 due to a decline in ovarian function (3).

Vasomotor prodrome, hot flashes, is the primary concern, with a third of menopausal women suffering from moderate to severe symptoms (4). Sleep disturbance, urogenital issues, mood swings and joint problems were also reported (5). These symptoms start a few years before menopause (perimenopausal stage) and continue for years after (6). Moreover, menopause results in several long-term consequences, such as osteoporosis and cardiovascular disease (1, 2).

Given that a significant number of women are expected to live one-third of their lives after midlife, menopause can adversely affect both women's quality of life and the healthcare system if left untreated. Therefore, women's health and quality of life are among the main concerns for healthcare management worldwide (7, 8).

Hormone replacement therapy (HRT) or Menopausal Hormone Therapy (MHT) stands out as the most cost-effective modality in relieving postmenopausal symptoms (7, 9, 10). Furthermore, both observational and randomised trial data consistently support the positive impact of HRT on reducing the risk of coronary heart disease (CHD), osteoporosis and overall mortality if initiated shortly after menopause (11). Up to 40%, 30%, 50% and 20% reduction in CHD, osteoporosis-related hip and spinal fractures and mortality was revealed due to the use of HRT (8, 11–14).

HRT lost its popularity in 2002 after the initial results of the Women's Health Initiative (WHI) trial (15), which presumed the risks of HRT outweighed the benefits (7, 16–18). For a significant number of younger menopausal women (age less than 60 years), the benefits of HRT in symptomatic relief outweigh any of the mentioned risks, according to the Endocrine Society Task Force report on hormone therapy (Lobo, 2013). Three menopause scientific societies, the North American Menopause Society (NAMS), the International Menopause Society (IMS) and the European Menopause and Andropause Society (EMAS) agreed about the beneficial role of HRT in treating symptomatic menopausal women (19).

HRT utilisation has been reported as low in several Arab-country settings, with substantial variation across studies included in this review (17). This low uptake could be attributed to several factors that are not well addressed in the Middle Eastern Region (20). Psychosocial and individual-level determinants (including attitudes and beliefs), in addition to the external influencers like information received and services provided, play a role in women's understanding and acceptance of HRT (7). All women have the right to be advised appropriately about the dramatic negative impact of menopause on their health, and need to be offered HRT in the safest and beneficial way (14, 21).

The regional evidence remains fragmented, with studies concentrated in a small number of countries and a limited synthesis of determinants of uptake across settings. A focused review is needed to consolidate what is known about barriers and facilitators to HRT utilisation in Arab countries, identify consistent patterns across studies, and summarise proposed strategies that are culturally appropriate and relevant for clinical practice and public health. Restricting the review to Arab countries is justified because shared linguistic and socio-cultural contexts may shape menopause discourse, health-seeking behaviour, and treatment preferences, and because region-specific synthesis can better inform locally relevant policy and service improvements.

The main aim of this systematic review was to investigate factors influencing the uptake and utilisation of HRT in the Arab Countries. Additional objectives were to assess the level of HRT knowledge in the region, whether any community-based interventions have been implemented, and their impact on increasing awareness and use of HRT.

## 2 Methods

This systematic review was registered with PROSPERO under registration number 2025 CRD420251007430 (22). Articles meeting the inclusion criteria were retained for data extraction. The following data were extracted from selected studies: authors/ publication year, study design, setting, sample size, age, data collection method, key findings, and literature recommendations, Supplementary Table S1.

### 2.1 Search criteria and critical appraisal

The methods used in this systematic review adhered to the Preferred Reporting Items for Systematic Review and Meta-analysis (PRISMA) reporting guidelines (23). Embase®, WHO IRIS, Cochrane Library of Systematic Reviews, CENTRAL, and Scopus were searched till 13 March 2025. The search included MeSH terms and free text within each database. Keywords included can be found in Supplementary Table S2 (Main keywords: Menopause; Menopause transition/ perimenopause; Hormonal therapy; Uptake; Utilisation; Use; HRT; Arab countries; Barriers; Factors; Hormone replacement therapy HRT; Knowledge; Perception; Attitude). The Arab countries included as keywords are Oman, Bahrain, Iraq, Algeria, Egypt, Jordan, Kuwait, Lebanon, Libya, Mauritania, Morocco, Palestine, State of, Qatar, Saudi Arabia, Somalia, Sudan, Syrian Arab Republic, Tunisia, United Arab Emirates (UAE), Yemen, Comoros, and Djibouti.

Two independent reviewers screened the studies on Covidence. Covidence was used to identify and remove duplicate records across databases using its automated deduplication function. We then undertook a manual verification step to identify potential residual duplicates by comparing titles, author lists, year of publication, journal, and, where available, DOI/unique identifiers, retaining the most complete record for screening. A third reviewer resolved any conflicts that arose during screening. Publications were reviewed through their titles and abstracts; if they met the inclusion criteria, they were nominated for full-text review. The Newcastle-Ottawa Scale (NOS), adapted for use in cross-sectional studies, was employed to assess the quality of individual studies and the risk of bias (24). Most articles successfully passed the quality and bias assessment; however, some have a high risk of bias, as shown in Supplementary Table S3.

### 2.2 Inclusion and exclusion criteria

This review included studies focused on women (aged 20 years and over) living in Arab countries who are using HRT to manage menopausal or perimenopausal symptoms. Eligible studies must compare HRT users to non-users within the same demographic, aiming to identify key factors. Only peer-reviewed studies published in English or Arabic were considered, including quantitative (e.g., cross-sectional, cohort), qualitative, and mixed-methods research conducted in clinical or community settings. Studies involving non-Arab populations, women outside the specified age range, or those not focusing on HRT for menopause were excluded. The primary outcomes included assessing the prevalence of HRT use, exploring barriers and facilitators to its utilisation, and proposing recommendations to support informed decision-making among Arab women. There are no restrictions on publication dates. There were no restrictions on the time of publication. The screening procedure was conducted using Covidence software, with two reviewers involved, and any disagreements were resolved by a third reviewer.

### 2.3 Data extraction

The extracted data were summarised in tables and graphs to allow for comprehensive comparison. Findings and gaps within the literature and the certainty of the evidence were assessed based on synthesis precision, the number of studies, and relevance to the study's questions. Extracted data included author names, country, study design, sample demographics and size, year of publication, and key findings.

## 3 Results

**Figure 1:**
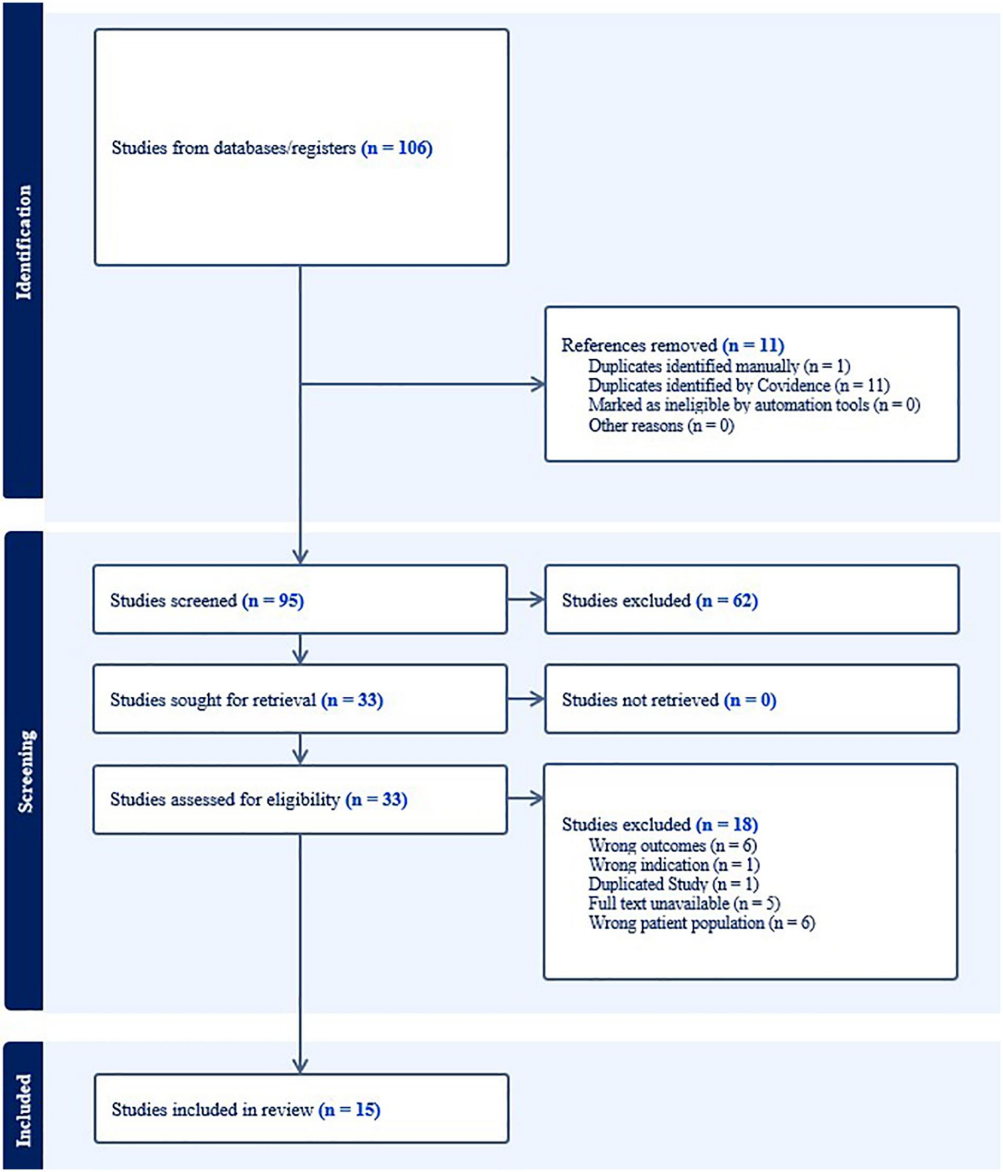
PRISMA flowchart from covidence showing the screening process and the number of included studies in this systematic review.

Out of 106 articles screened, 15 studies were cross-sectional and met the inclusion criteria out of 33 papers with full text screened. The PRISMA flow chart of the search and process of selecting the references is shown in Figure 1. Sample sizes ranged from 29 to 591 participants. Detailed characteristics and key findings of the included studies are presented in Table 1.

**Table 1:** Study characteristics and Key findings of the 15 included studies.

No.	Author (Year)	Country	Study design	Sample size	Population (Age group)	Key findings on HRT
1	Shahzad et al. (2021) (20)	UAE, Dubai	Cross-sectional	591	≥40 years	- HRT use: 6% - HRT knowledge: 13.7%
2	Hamid et al. (2014) (8)	UAE, Al Ain	Cross-sectional	177	≥40 years	- HRT use: 7% - HRT knowledge: 27%
3	Ibrahaim & Hussein (2016) (10)	UAE, Abu Dhabi and Sharjah	Cross-sectional	220	20–70 years	- HRT use: 48% - High awareness
4	Jassim & Al-Shboul (2009) (28)	Bahrain	Cross-sectional	260	30–64 years	- HRT use: 3.9% - HRT knowledge: 60%
5	Loutfy et al. (2006) (25)	Egypt, Alexandria	Cross-sectional	450	50–59 years	- HRT use: 0% - HRT knowledge: 9.3%
6	Smail et al. (2020) (31)	UAE, Dubai	Cross-sectional	497	30–64 years	- HRT use: 9% - HRT knowledge: 40.56%
7	Mustafa & Sabir (2012) (30)	Iraq, Erbil	Cross-sectional	500	40–60 years	- HRT use: not mentioned - HRT knowledge: 13.6%
8	Salame et al. (2020) (26)	Lebanon, Beirut	Cross-sectional	123	≥40 years	- HRT use: 29.8% - HRT knowledge: 64%
9	Albaqami et al. (2023) (29)	Saudi Arabia, Taif	Online cross-sectional	383	40–65 years	- HRT use: 6.2% - HRT knowledge: 16.4%
10	Alshogran et al. (2021) (34)	Jordan, Irbid	Cross-sectional	450	20–40 years	- HRT use: Not mentioned - HRT knowledge: Majority unaware
11	AlSwayied et al. (2024) (36)	Saudi Arabia	Cross-sectional	29	40–64 years	- HRT use: Low interest - HRT knowledge: some basic awareness
12	Bakarman & Abu Ahmed (2003) (32)	Saudi Arabia, Western	Cross-sectional	300	35–70 years	- HRT use: 5% - HRT knowledge: 26.7%
13	Aladhab and Alabbood (2021) (37)	Iraq, Basrah	Cross-sectional	500	41–65 years	- HRT use: 18.2% - HRT knowledge: 21%
14	Tosson et al. (2014) (27)	Egypt, Assiut Hospital	Cross-sectional	99	≥45 years	- HRT use: 23% - HRT knowledge: 38%
15	Albeitawi et al. (2024) (35)	Jordan	Cross-sectional	566	45–65 years	- HRT use: 14.3% - HRT knowledge: Not mentioned

**Table 2:** The frequency (number, *n*) and percentage (%) of factors that influence knowledge and the uptake of HRT in the studies included, *n* = 15.

Positive influencing factors/Facilitators	Frequency (*n*)	Percentage (%)
Higher educational level	9	60%
Employment	7	46.6%
Involvement of health care providers/ health care system	6	40%
Presence of severe symptoms	3	20%
High economic status	3	20%
Age	2	13.3%
Physicians’ positive knowledge and attitudes towards HRT and menopause	2	13.3%
Positive attitude and views on ageing and menopause	2	13.3%
Believes in HRT benefits	2	13.3%
Marital Status	1	6.6%
Regain feminisation	1	6.6%
Prevent medical complications	1	6.6%
Negative influencing factors/Barriers	Frequency (*n*)	Percentage %
Concerns about HRT safety and side effects	6	40%
Beliefs and culture influence	5	33.3%
Believe in alternatives/ herbal products.	4	26.6%
Inadequate menopause knowledge	3	20%
Cost and socioeconomic status	2	13.3%
Medication not offered or discussed by doctors	2	13.3%
Negative influence of the media	1	6.6%
Lack of knowledge about HRT	1	6.6%
Other medical problems	1	6.6%
Education level [illiterate]	1	6.6%

Four studies were conducted in the UAE, three in Saudi Arabia, and two each in Egypt, Iraq, and Jordan, with one study each in Bahrain and Lebanon. No studies that fit the inclusion criteria were found in the other Arab countries.

### 3.1 HRT utilisation

There is some variability in the uptake of HRT, which was relatively low, with less than 20% of the participants in 11 of the included articles. None of the participants from Alexandria in Egypt had ever used HRT (25), and Bahrain reported only 3.9% of the participants. The highest uptake among the studies was reported in the UAE: Abu Dhabi and Sharjah studies at 48% of 220 participants, followed by Lebanon, 29.8% and Egypt 23% (10, 26, 27). The included Gulf states (UAE, Saudi Arabia, Bahrain) had an average use of approximately 10% (3.9%–48%).

### 3.2 HRT knowledge

Most studies reported <30% HRT knowledge, except in Lebanon (26) at 64% (highest) and Bahrain (28): 60% (but only 3.9% usage), likely due to stronger healthcare provider involvement. High knowledge did not always show high use of HRT. HRT knowledge was notably low in Egypt (9.3%), Iraq (13.6%), UAE-Dubai (13.7%), and Saudi Arabia, Taif (16.4%) (20, 25, 29, 30). The UAE showed both extremes, 13.7% knowledge (31) vs. 48% use (10). Table 1 provides more details on the uptake and HRT knowledge.

### 3.3 HRT facilitators and barriers

**Figure 2:**
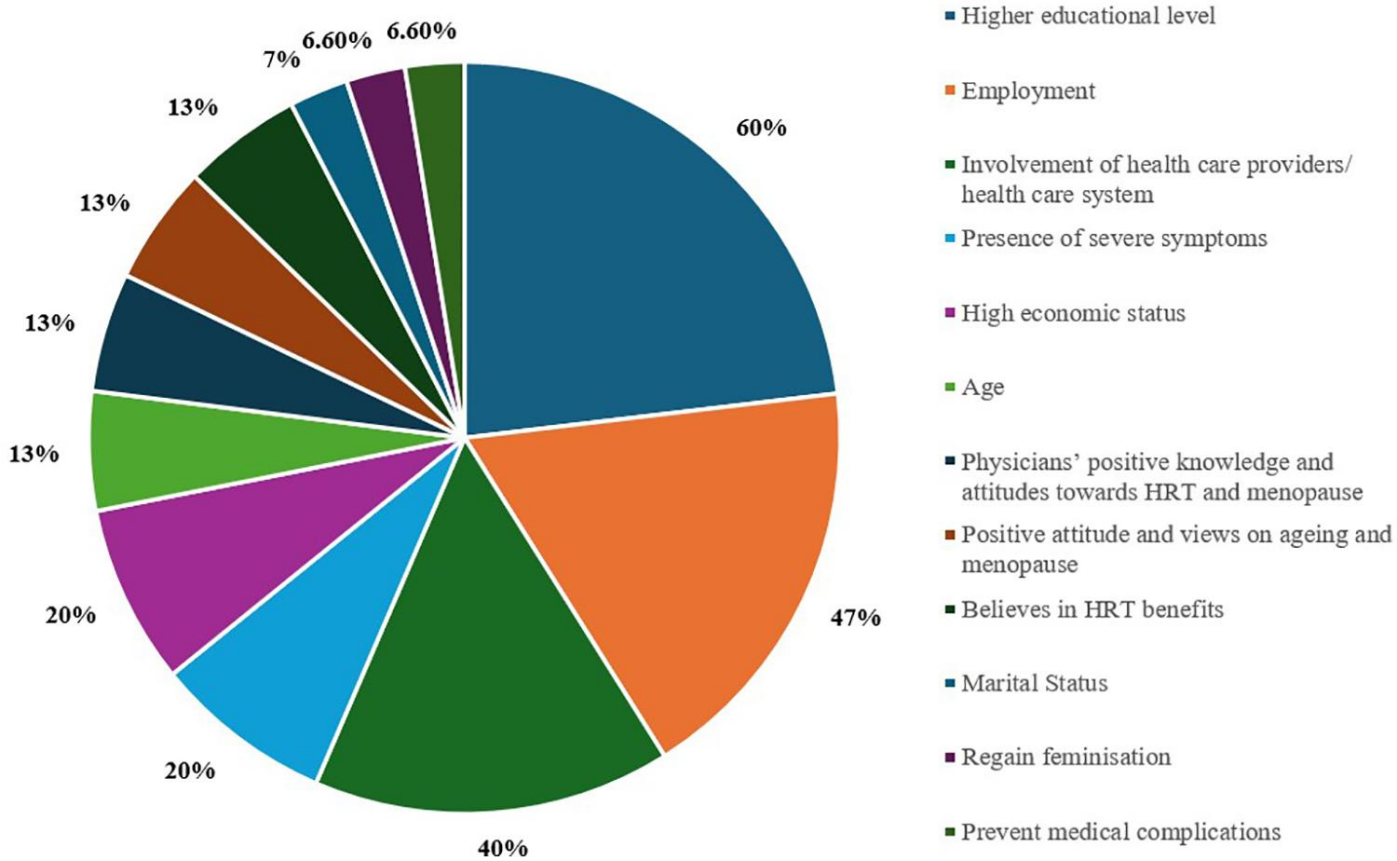
Percentage (%) of facilitators for the knowledge and the uptake of HRT in the included studies, *n* = 15.

**Figure 3:**
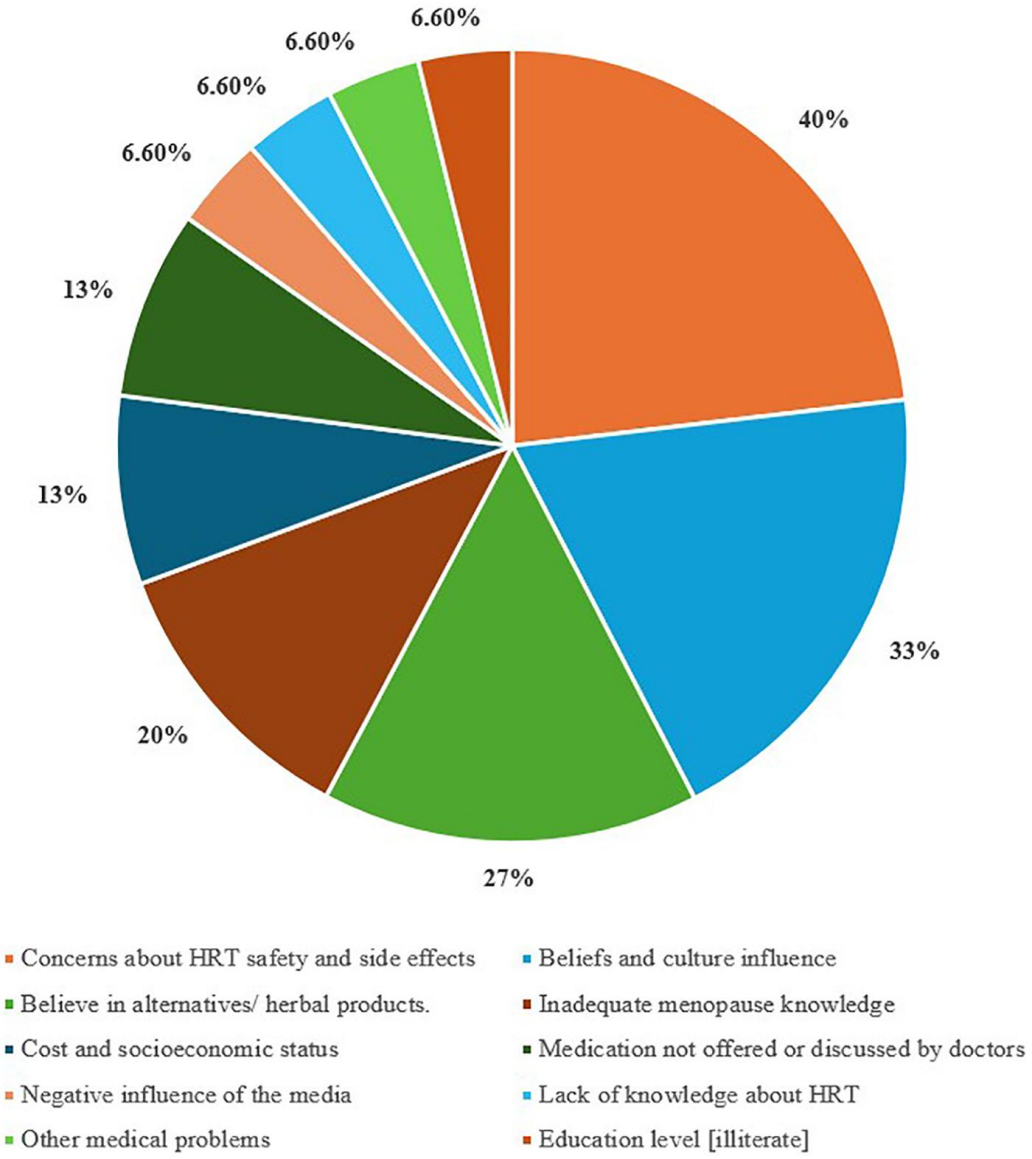
Percentage (%) of barriers to the knowledge and uptake of HRT in the included studies, *n* = 15.

The study identified key influencing factors for HRT use, including educational/professional status, healthcare system engagement and positive knowledge and attitudes of physicians. Beliefs in alternative treatments were mentioned by four studies (8, 20, 25, 32), while menopausal knowledge was reported by three (10, 27, 33). Five studies reported on cultural considerations, and additional studies mentioned the safety and side effects associated with HRT uptake (Supplementary Table S1). Severe symptoms, and increased economic status were mentioned by three studies each. Age, beliefs in HRT benefits, and positive attitudes towards menopause were mentioned by seven studies. Other factors, including media influence, cost, sudden menopause, lack of symptoms, not advised by physicians, access concerns, and association with body shape, were also reported. These findings highlight the complex interplay of socio-cultural, economic, and healthcare factors influencing HRT acceptance in Arab countries. Figures 2, 3 illustrate the factors that act as barriers and facilitators reported in the 15 included studies.

Studies reported critical gaps such as low HRT use in Egypt, Bahrain, and parts of the UAE, which is often linked to cultural preferences for herbal alternatives or lack of physician counselling (25, 32, 34). Higher use in Lebanon, Iraq, and UAE-Abu Dhabi (10, 26, 35), where physician recommendations and severe symptoms drove uptake.

Media dominance as a primary information source in 6 of the included studies (e.g., UAE-Dubai, Saudi Arabia) correlated with misconceptions (20, 33), while physician-led education improved knowledge (10).

#### 3.3.1 Facilitators

The level of education and employment was mentioned in 60% of included articles as a significant positive influencer, followed by employment (46.6%). Higher economic status is also positively associated with better knowledge and attitudes towards HRT, 20% (8, 25, 26, 35).

Adequate HRT knowledge and uptake are highly influenced by having good basic knowledge of menopause, *p*-value <0.001 (10, 20). Conversely, low awareness of menopause and associated issues impacts coping strategies and attitudes toward HRT (35, 36). However, most women in this review had inadequate knowledge of basic menopause.

The influence of health care providers and health systems was identified in 40% of the included literature. The percentage of women who received medical consultation after menopause ranges between 9% and 16% in some studies (8, 20, 25, 27), and only Lebanese and Iraqi women had a better chance to discuss menopause with their physicians, 36.7% and 49% respectively (26, 30). Physicians’ positive knowledge and attitudes towards HRT influenced its uptake (10, 26). The study conducted in the UAE by Ibrahimi & Hussein (2016) found that HRT knowledge was associated with the availability of good health clinics and greater opportunities to consult a medical specialist in urban areas than in rural areas. HRT knowledge was significantly higher in Abu Dhabi (68%) compared to Sharjah (39%) (*p* < 0.05). Abu Dhabi offers more qualified doctors with stronger communication skills and easier access to primary care clinics.

Symptom severity significantly impacted attitudes toward HRT. Women with more severe climacteric symptoms had more positive attitudes toward HRT compared to those with milder symptoms (8, 20, 27). Awareness of HRT's benefits in relieving symptoms also encouraged use (10, 26, 35).

Other less frequently mentioned factors included age, BMI, regaining feminisation, and marital status. The UAE-Al Ain study identified age >50 and BMI <30 as significant predictors of HRT uptake (8), while the Egypt-Alexandria study found younger women had more positive attitudes (25).

#### 3.3.2 Barriers

Concerns about HRT risks were expressed in 6 studies (40%). Beliefs and conservative Arab culture were mentioned in 33.3% of the studies as negative influencers against seeking menopause medical treatment. In a study conducted in Egypt, many women view menopause as a private issue that should not be discussed with others and as a normal milestone of the ageing process, which does not require medical intervention (25). Arab knowledge of menopause treatment is significantly lower than that of non-Arabs (*p* < 0.01), and this could be attributed to the conservative nature of the region (20, 30). The inadequate knowledge of menopause was mentioned in 20% of the included studies as a barrier to HRT use.

The preference for natural or alternative products over HRT was mentioned in 26.6% of the studies. Women in Egypt, Iraq, and the UAE preferred natural remedies for menopause symptoms (25, 32, 37). For example, two-thirds of Emirati women in Smail et al.'s study in 2020, 41% in Shahzad et al.'s in 2021, and 58% in Hamid et al.'s in 2014 favoured alternatives over HRT. 24% in Smail's believed HRT's risks outweighed its benefits.

Cost was a barrier in Egypt (25), while the lack of medication availability hindered use in Jordan (36). All positive and negative factors that influence HRT uptake, along with their numbers and percentages, were summarised in Table 2.

**Table 3:** Key recommendations from the included studies to enhance HRT use.

Study (Year)	Key recommendations
Shahzad et al. (2021) (20)	Patient-centred consultations; multisector collaboration (GPs + community workers); educational programme needed.
Hamid et al. (2014) (8) + Tosson et al. (2014) (27)	More research on physician menopause perception.
Ibrahim & Hussein, (2016) (10)	Community-based campaigns to raise awareness of treatment availability.
Jassim & Al-Shboul (2009) (28)	Research is needed on alternative therapies and awareness among different ethnic groups.
Salame et al., 2020 (26)	Physicians should provide patients with information and counselling.
Alshogran et al., 2021 (34)	Educational programmes for women on pharmacological and non-pharmacological approaches; enhance scientific communication on multimedia channels
AlSwayied et al. (2024) (36)	Holistic approaches (self-care + family support); online Arabic resources.
Bakarman & Abu Ahmed (2003) (32)	Public media campaigns and direct education via medical professionals.
Tosson et al. (2014) (27)	National education programs to combat stigma; focus on doctor attitudes.
Albeitawi et al. (2024) (35) + Smail et al., (2020) (31) + Albaqami et al., 2023 (29) + Aladhab R & Alabbood M 2021 (37)	Awareness campaigns on HRT safety and benefits, especially through health professionals.

### 3.4 Recommendations

The reviewed studies consistently called for a multifaceted approach to improve HRT awareness and utilisation in Arab countries (Table 3). A prominent recommendation centred on healthcare system reforms, with multiple studies advocating for patient-centred consultations (20) and enhanced physician engagement in menopause management (26). Researchers emphasised that physicians should proactively discuss HRT options and provide evidence-based counselling, as inadequate provider communication was identified as a major barrier (27, 36). Several studies specifically recommended training programmes for healthcare providers to address gaps in menopause knowledge and combat prevailing stigmas (8, 27).

At the community level, studies strongly recommended public awareness campaigns to improve knowledge about menopause and HRT (10, 33). These should include culturally adapted educational programs covering both pharmacological (HRT) and non-pharmacological approaches, delivered through multimedia platforms for wider reach (32, 35). The need for targeted messaging was highlighted, particularly to address widespread misconceptions about the risks and benefits of HRT (29, 38). Additionally, researchers suggested developing Arabic-language digital resources and online support groups to improve accessibility and provide reliable information (37).

The studies also emphasised holistic strategies incorporating family and social support systems, recognising the cultural importance of collective decision-making in Arab societies (37). Further research was recommended to explore alternative therapies and ethnic-specific perceptions of menopause to better address diverse patient needs better (34).

## 4 Discussion

The included studies showed that HRT uptake is lower in Arab countries, and several factors were identified as key drivers of that lower uptake. These factors included educational and professional status, involvement in the healthcare system, beliefs in alternative treatments, basic knowledge of menopause, conservative culture, economic status, concerns about side effects, media influence, age, Body Mass Index (BMI), and cost.

Advanced education and employment were the highest positive factors trending in this study, which agrees with (16, 39). Educated women are likely to be aware of available treatment with better knowledge of the benefits and risks of HRT, allowing them to negotiate and make informed decisions (16, 34, 40). Consistently, economic status was also an influencing factor, which agrees with previous studies (6, 16, 41). In fact, inequity in receiving health services makes less privileged women cannot ask for higher-quality health measures (42). The study found that the knowledge of women regarding HRT is very poor compared to Africa, Asia and Europe (9, 17–19, 43, 44).

The pattern observed in Arab-country studies, low utilisation alongside limited clinician-led counselling and strong influence of non-clinical information sources, aligns with findings from other regions, indicating that treatment decisions are closely linked to knowledge, risk perceptions, and the availability of trusted medical advice (9, 16, 17, 39, 45). In population-based and survey studies outside the region, utilisation and attitudes likewise vary by socioeconomic position, access to care, and whether clinicians initiate discussions about menopause management (6, 38–40, 44). However, the included Arab-country studies more frequently emphasised socio-cultural norms around discussing menopause, preference for herbal or “natural” remedies, and menopause being framed as a private or “normal” ageing process as prominent barriers, which may differ in salience across settings (25, 31, 36, 46). These comparisons underscore that while determinants such as education and clinician engagement are common across contexts, culturally mediated beliefs and communication norms may be particularly important in shaping HRT uptake in Arab countries.

In this systematic review, most women did not receive medical advice after menopause. This could be related to the accepting attitude of physicians towards HRT and the perception of menopause as a natural process not requiring treatment, except in Lebanon, modern cities in Abu Dhabi, where highly qualified physicians, with better communication, and easy access to the health system, were available (10, 26). The result agrees with other previous studies (16, 45).

However, variation between different regions in HRT prescriptions could be attributed to physicians' qualifications, experience in the field, being updated in HRT evidence and their personal perception, which significantly impacts their confidence to discuss this issue (1, 45, 46). Routine prescription of HRT could be the main reason for high HRT uptake seen in the Lebanon study, which is consistent with a study in Spain, where 62% of women took HRT on doctors' advice (47). Even though women are not able to make self-decisions in managing menopausal symptoms without physicians' guidance (40, 48). Physicians should not take the lead either to encourage or to build fear in women with regard to HRT; the women should decide after receiving appropriate advice. Women need to balance between tolerating menopause symptoms and improving their quality of life (49). The Consensus Conference in Italy agreed that women have the right to receive this knowledge (49, 50) and that this can be done through routine health visits or women's preventative programmes like mammograms and pap smear screening (34).

In our study, only two articles where physicians were the primary source of menopause education, where the hormone uptake was the highest (10). Compared to the 2011 systematic review, 47% of their participants received the knowledge from the medical team (17). These findings emphasise the important role of physicians in promoting HRT uptake. In Lebanon, despite the uptake being acceptable, the main source of information was the media, which had a negative impact on the avoidance or cessation of routinely prescribed HRT, often administered without proper consultation (26). Consistently, in West of SA and Jordan, media represented 28% and 19% of the information, respectively, but no considerable knowledge was reported about HRT (33). This result is in line with a strong influence of negative information in the media, which prevents Spanish women from starting or continuing to use HRT (47).

The presence or absence of severe symptoms plays an important role in the level of HRT uptake in this study. Women may also be less able to recognise menopause symptoms compared to those in different geographical areas (51). The Middle East, where Arab women belong, is rated as having less than in Europe and America (1, 7, 9, 18, 19, 44, 45, 52).

The study also showed that many women view menopause as a natural ageing process, like puberty, and they are not accepting discussion about it. Avoiding the talk about menopause is strongly related to culture and religion (2, 48).

The review showed that 40% of the studies included addressed the risk associated with HRT uptake, which could be attributed to low HRT awareness. In fact, concerns regarding HRT risks impact women's decisions in using HRT, especially after the WHI study (1, 16, 53). The premature results of the WHI have made a negative footprint with misleading information and exaggerated anxiety towards HRT use (18, 19, 47). However, follow-up and re-analysis of the WHI study emphasise the importance of considering the age when starting the HRT(<60 years), time since menopause (window of opportunity, less than 10 years), and hormone regimen to be used (individualised according to risks) as crucial factors in offering HRT (7, 19) to mitigate the relatively small risk associated with the use of HRT tablets in the elevated risk of breast cancer, increased risk of stroke and heart disease, if used after the age of 60 years and with prolonged use (54). All women have the right to be advised appropriately about the dramatic negative impact of menopause on their health, and need to be offered HRT in the safest and most beneficial way (14, 21).

No direct intervention of the community was found in this study. However, a community-based campaign in educating Bahraini women about osteoporosis has a strong impact in increasing their awareness of the role of HRT in preventing and treating osteoporosis when compared with other Asian countries (10). Another unintended intervention was seen in the Lebanese health system, where women routinely receive HRT, even without adequate discussion on risks and benefits, which has a positive effect on promoting HRT uptake. This contradicts the well-established interventions seen in the UK, which involve two key influencing factors: education and the healthcare system. It is agreed on the importance of enhancing health care providers' ability to manage menopause in a standardised approach by releasing the first menopause guidelines in 2015 by the National Institute for Care and Excellence (NICE) (55). The second intervention was the addition of menopause as a mandatory topic to the national school curriculum in 2019, as part of the Relationship and Sex Education (RSE) subject, which must be taught in England National Schools (55).

### 4.1 Strengths

The study has several strong points; it is the first systematic review to address the critical gap in the literature on the assessment of HRT uptake enablers and barriers in Arab countries. It provides a comprehensive overview of the global and local literature, highlighting the need for regional studies to analyse variations in the factors influencing knowledge and attitudes towards HRT across different societies. Additionally, it provides a rationale for community-based interventions, offering women adequate information about menopause consequences and the availability of treatment to enhance the quality of care. It also guides physicians to understand the viewpoints of women towards HRT and empower their role in treatment decisions. Our results will enhance healthcare providers' understanding of demographic factors and cultural influences, and their role in providing appropriate assessments and consultations for each woman, as well as in offering HRT when required. The perceptions and attitudes of middle-aged women and physicians are equally important; they were assessed simultaneously in this review to provide a broader picture of the topic and facilitate further correlation between factors affecting both views.

### 4.2 Limitations

It is important to mention that this review has several limitations. First, there is diversity between the studies in terms of sample size, clinical settings, and main objectives. In addition to the use of non-standardised methodology and investigational tools in assessing the level of knowledge about short- and long-term benefits and risks of HRT. Such discrepancies made the comparability of findings more difficult across studies. Most of the included studies relied on qualitative cross-sectional studies, which results in recall and respondent bias. Finally, the lack of data on HRT in many parts of the region directly impacted the generalisation of the systematic review's findings.

## 5 Conclusion

This systematic review reveals a concerning gap in menopausal healthcare across Arab countries, where women remain largely uninformed about hormone therapy as an effective treatment option. The findings demonstrate how this knowledge deficit contributes to low HRT utilisation, leaving many women to endure menopausal symptoms without access to proper medical guidance. More importantly, this work highlights a critical literature gap regarding menopause management in Arab countries, while simultaneously laying the groundwork for developing culturally appropriate interventions.

Across included studies, higher education/employment, symptom severity, and clinician counselling were the most consistently reported facilitators, while reliance on non-clinical information sources and preference for alternative remedies were common barriers. Strengthening clinician–patient communication and implementing culturally adapted community education were the most frequently recommended strategies to support informed decision-making and improve menopause care in the region.

## Data Availability

The original contributions presented in the study are included in the article/Supplementary Material, further inquiries can be directed to the corresponding author.
