# Supplementary material for: Utilisation of hormone replacement therapy in Arab countries: a systematic review
Source: Front Glob Womens Health. 2026 Feb 9;7:1722268. doi: 10.3389/fgwh.2026.1722268 (PMC12926417; doi:10.3389/fgwh.2026.1722268)
Supplement: Supplementary file 1 [file Datasheet1.docx]

Supplementary Table 1: Data Extraction Table for 15 Included Studies.

| no | Authors /year | Study design & Data Collection Method | Setting | Age Group & Sample Size | Key findings | Conclusion/Recommendations |
| --- | --- | --- | --- | --- | --- | --- |
| 1 | (Shahzad et al., 2021) | Cross-sectional  Data collection: Direct interview | UAE /Dubai | 40 years and over  Sample size: n=591 | Facilitators:   - Higher educational level - Involvement of health care providers/ health care system - Severe symptoms   Barriers:   - Inadequate menopause knowledge - Beliefs in alternatives/herbal products. - Beliefs and culture influence   HRT use: 6%  Sat for medical counselling: 16%  Menopausal knowledge: good to fair knowledge in 66%  HRT knowledge: 13.7%  Main source of information: Media | Conclusion   - GPs play a vital role in diagnosing menopause and providing adequate information on HRT - The knowledge about menopause and HRT is consistent with each other - Most health care providers do not discuss HRT with patients, resulting in poor awareness   Recommendations:   - Patient-centred consultations on the pros and cons of HRT should be encouraged. - Multisector collaboration in health teams, including GPs and community workers, to provide postmenopausal women with good quality of care. - Tailoring an educational program for intervention (pre-post-menopausal) to educate women. |
| 2 | (Hamid et al., 2014) | A clinic-based cross-sectional  Data Collection: Interviewer administered survey | UAE / Al Ain city) | >40 years  Sample size: n=177 | Facilitators   - Higher educational level - Involvement of health care providers/ health care system - Severe symptoms - Increased economic status   Barriers:  Believe in alternatives/ herbal products.  HRT use 7%  Sat for medical counselling: 13%  menopausal knowledge 33%  HRT knowledge:  27%  Main source of information: Media | Conclusion:  No formal education about sexual and reproductive health, neither in schools nor in clinics.  Recommendations:   - Importance of considering menopause symptoms as a problem that requires attention and management. - More qualitative studies are needed to investigate how women and physicians perceive menopause and highlight factors influencing HRT prescriptions. |
| 3 | (Ibrahim and Hussein, 2016) | Cross-sectional study  Data Collection: Interview | UAE / Abu Dhabi and Sharjah | 20-70 years  Sample size: n=220 | Facilitators   - Higher educational level - Employment - Involvement of health care providers/ health care system - Physicians’ positive knowledge and attitudes towards menopause - Believes in HRT benefits   Barriers:   - Inadequate menopause knowledge - Concerns about HRT safety and side effects   HRT use: 48%  Sat for medical counselling: high  menopausal& HRT knowledge: high level of awareness  Main source of information: physicians | Conclusion:  Medical counselling stimulates understanding of treatment options and guides women’s decisions regarding HRT.  Recommendations:  Knowledge of the long-term consequences of menopause should be spread through community-based campaigns to increase public awareness of the availability of treatment. |
| 4 | (Jassim and Al-Shboul, 2009) | Cross-sectional study  Data Collection: Interview-administered questionnaire | Bahrain / five governorates: Attended primary health care centres | 30-64 years  Sample size: n=260 | Facilitators:   - Higher educational level - Employment   Barriers  Not mentioned  HRT use: 3.9  Sat for medical counselling: not mentioned.  menopausal& HRT knowledge: 60%  Main source of information: not mentioned | Conclusion:  Knowledge of Bahraini women's pre-menopause and HRT on average.  Poor knowledge about the consequences of menopause on cardiovascular disease.  Recommendations:   - Further studies are needed to estimate the level of awareness of menopause among different ethnic groups. - Research on alternative therapies - Improving research quality in studying the risks and benefits associated with HRT and its long-term consequences. - The awareness of the safety of using a low dose of oestrogen is needed |
| 5 | (Loutfy et al., 2006) | Cross-sectional study  Data collection:  a qualitative and a community-based, quantitative, cross-sectional survey | Egypt / Alexandria governorate | 50-59 years  Sample size: n=450 | Facilitators:   - Higher educational level - Marital status - Increased economic status   Barriers:   - Cost - Beliefs and culture influence - Believe in alternatives/ herbal products. - Concerns of HRT safety and side effects   HRT use: 0%  medical counselling: 11.1%  menopausal knowledge: 38.4  HRT knowledge: 9.3%  Main source of information: Media | Conclusion:  Most women are aware of perimenopausal changes.  Recommendations:  Utilising media and GPs to spread the knowledge related to menopause issues and management is crucial. |
| 6 | (Smail et al., 2020) | Cross-sectional study  Data Collection: Questionnaire Interview | UAE /Dubai | 30-64 years  Sample size: n=497 | Facilitators:   - Higher educational level - Employment - Age <50y   Barriers:   - Believe in alternatives/ herbal products. - Concerns about HRT side effects   HRT use: 9%  Sat for medical counselling: not mentioned.  menopausal & HRT knowledge: 40.56  Main source of information: Media. | Recommendations  Advancing community knowledge to improve Menopause management |
| 7 | (Mustafa and Sabir, 2012) | Cross-sectional study  Data collection: Closed-ended self-administered questionnaire. | Iraq /Erbil city | 40-60 years  Sample size: n=500 | Facilitators   - Higher educational level - Involvement of health care providers/ health care system - Employment   Barriers:  Beliefs and culture influence  HRT use: not mentioned  Sat for medical counselling: 49%  Menopause knowledge 56.6%  HRT knowledge: 13.6  Main source of information: not mentioned. | Conclusion:  The majority considered menopause a normal phase, and their unawareness of HRT is low. |
| 8 | (Salame et al., 2020) | A clinic-based cross-sectional  Data Collection: Questionnaire | Lebanon / Beirut | 40 years and above  Sample size: n=123 | Facilitators:   - Employment - Involvement of health care providers/ health care system - Physicians’ positive knowledge and attitudes towards menopause - Increased economic status. - Believe in HRT benefits   Barriers:  Media negative influence  HRT use: 29.8  medical counselling: 36.7%  Menopausal Knowledge: not mentioned  HRT knowledge: 64%  Main source of information: Media | Conclusion:  Physicians prescribe HRT without discussing long-term effects.  Recommendations:   - Doctors should provide women with an overview of evidence-based information in HRT to avoid misleading information. - Managing menopause through adequate counselling is essential. |
| 9 | Albaqami et al., 2023 | Cross sectional  Data collection: Online-administrate Questionnaire | Taif, Saudi Arabia | 40-65 years  Sample size: n=383 | Facilitators:  Employment  Barriers:  Not mentioned  HRT Use:6.2%  Current use 1.8  Previous use 4.4%  Sat for medical counselling: not mentioned  Menopausal knowledge: not mentioned  HRT knowledge: 16.4%  Main source of information: media | Conclusion:  Not being informed about HRT is the main reason for not using it.  Recommendation:  Community awareness programmes are recommended to facilitate the knowledge and use of HRT |
| 10 | (Alshogran et al., 2021) | Cross-sectional study  Data Collection:  Questionnaire through interview | Irbid, Jordan | 20-40 years  Sample size: n=450 | Facilitators:   - Level of education (college or over) - Positive views on menopause and ageing process   HRT use: not mentioned  Sat for medical counselling: not mentioned  Menopause knowledge more than 50% had accurate knowledge  HRT knowledge:  Majority did not know the role of MHT  Main source of information:  57% family and friends  19% media  13% healthcare provider  11% other | - There is knowledge about menopause, but less knowledge about MHT.   Recommendations:   - Educational programmes are needed about menopause and health consequences. - Enhance women’s understanding of various pharmacological and non-pharmacological approaches aimed at addressing menopausal symptoms and preventing related complications. - Enhance the scientific communication of healthcare providers as sources of information through magazines and websites. |
| 11 | (AlSwayied et al., 2024) | Semi-structured interviews: qualitative study | Saudi arabia | 40-64 years  Sample size: n=29 | Facilitators:   - Positive attitude about menopause   Barriers:   - Experience with sudden or treatment-induced menopause - Cultural norms and social stigma - Perceived risk of cancer and lack of knowledge of treatment options   HRT use: most considered medical intervention unnecessary – little interest in HRT  Sat for medical counselling:  No advice from doctors  Menopause knowledge more than 50% had accurate knowledge  HRT knowledge:  Some had basic awareness  Main source of information:  Did not discuss with GPs – resorted to online resources discreetly | - Attitudes have shifted about menopause to a more positive one - Role of family and support to women is crucial during menopause - Advocating for a holistic approach, including self-care practices, can help reduce stigma in conversations with doctors. - There is a need to improve knowledge of HRT - More focus on primary care and dedicated clinics.   Recommendations:   - Arabic menopause management and treatment health information on online sources are needed - Sharing personal stories to show similar experience about women and establishing online support groups can be helpful. - Healthcare providers need to proactively ask women about menopause and educate about HRT through holistic approach. |
| 12 | (Bakarman and Abu Ahmed, 2003) | Cross sectional study  Data collection: Interview by questionnaire | Western Saudi Arabia | 35-70 years  Sample size: n=300 | Facilitators:   - Educational level - Age - Occupation/ Employment level   Barriers:   - No symptoms - Fear of side effects - Not offered by doctors - Lack of knowledge about HRT and use - Medical problems   HRT use: 5% use - 39% unwilling to use  Sat for medical counselling:  Not mentioned  Menopause knowledge not mentioned  HRT knowledge:  2/3rds not aware of HRT- only 80 (26.7%) females were   - Highest awareness were among college level (60%) - Most aware group was nurses (77%); teachers (52%) - Higher among females still menstruating   Main source of information:  Media (28.8%); doctors (27.5%); friends (13.8%) | - 46% of the sample interviewed were willing to use HRT after increased knowledge and awareness - Highly significant relationship between awareness and willing to use HRT – which directs to making better decisions on contributing risk factors of lack of information and physicians’ recommendations.   Recommendations   - Provide a basic understanding of HRT risks and benefits of usage - Best methods of disseminating information is through public media, school and direct education through medical professionals. |
| 13 | (Aladhab and Alabbood, 2021) | Cross sectional study  Data Collection: Self-administered questionnaire (interviews) | Basra Iraq | 41-65 years  Sample size: n=500 | Facilitators:  Not mentioned  Barriers:  Not mentioned  HRT use: 18.2% received HRT  Sat for medical counselling:  20.6% regarding menopausal symptoms.  Menopause knowledge all had some knowledge – menopausal symptoms knowledge 68.8%  HRT knowledge:  21% aware of HRT  Main source of information:  Not mentioned | - Most common symptom was hot flashes and second most common was back or joint pain - Significant relationship between BMI and age [higher BMI had delayed menopause]. Obese women have more estrogen   Recommendation:   - Increase awareness about symptoms and HRT benefits |
| 14 | Tosson et al., 2014 | Cross-sectional study  Data collection:  Structured questionnaire | Egypt Assiut University Hospital | Above 45 years  Sample size: n=99 | Facilitators:   - Unpleasant severe symptoms - Prevent medical complications - Regain feminization - Staff role/ Health providers' involvement   Barriers:   - Educational status (illiterate) - Social relations in arab communities - Socioeconomic status and access to information - Concern about body shape, age, and a negative view of menopause   HRT use: 23% used HRT  Sat for medical counselling:  24% sat for HRT discussions  Menopause knowledge 72% knew about specifics  HRT knowledge:  38% heard about HRT  Main source of information:  Friends and relatives (40.4%); doctors (38%); reading material, TV, Radio (10%) | - Health staff in the hospital succeeded in changing the previous perception of women on HRT and usage in this study. - Doctors and media are inadequately discussing HRT   Recommendations:   - Future studies should study the attitude and knowledge of doctors - Education programme should be started to spread knowledge about menopause and HRT |
| 15 | Albeitawi et al., 2024 | Cross sectional descriptive  Data collection:  Face-to-face and self-reported questionnaire | Jordan | 45-65 years  Sample size: n=566 | Facilitators:  Not mentioned  Barriers:   - Side effects - Medication not available   HRT use: 14.3% used  Sat for medical counselling:  45.9% about menopausal symptoms  Menopause knowledge not mentioned  HRT knowledge:  Not mentioned  Main source of information:  Not mentioned | - Less than half of the women in this study went to medical professionals about menopausal symptoms - Gap in HRT awareness and there are concerns of availability of the medication   Recommendations:   - Awareness of benefits, safety, and risks of HRT should be spread. - Awareness campaigns and health professionals play major role in HRT awareness and usage. |
