# Supplementary material for: Utilisation of hormone replacement therapy in Arab countries: a systematic review
Source: Front Glob Womens Health. 2026 Feb 9;7:1722268. doi: 10.3389/fgwh.2026.1722268 (PMC12926417; doi:10.3389/fgwh.2026.1722268)
Supplement: Supplementary file 2 [file Datasheet2.docx]

Supplementary Table 2: Database and Corresponding Search String Used in the Systematic Review.

| **Database** | **Search String** |
| --- | --- |
| **Ovid (Medline, Embase, APA PsycInfo, Global Health, Maternity and Infant Care, HMIC)** | (perimenopause OR menopause) AND ("hormone therapy" OR hrt) AND (knowledge OR attitudes OR perception) AND (afghanistan OR bahrain OR djibouti OR egypt OR iran OR iraq OR jordan OR kuwait OR lebanon OR libya OR morocco OR palestine OR oman OR qatar OR pakistan OR "saudi arabia" OR somalia OR sudan OR syrian OR tunisia OR "united arab emirates" OR yemen) |
| **PubMed** | (perimenopause OR menopause) AND ("hormone therapy" OR hrt) AND (knowledge OR attitudes OR perception OR utilisation) AND (afghanistan OR bahrain OR djibouti OR egypt OR iran OR iraq OR jordan OR kuwait OR lebanon OR libya OR morocco OR palestine OR oman OR qatar OR pakistan OR "saudi arabia" OR somalia OR sudan OR syrian OR tunisia OR "united arab emirates" OR yemen) |
| **Scopus** | TITLE-ABS-KEY ( perimenopause OR menopause ) AND TITLE-ABS-KEY ( "hormone therapy" OR hrt ) AND TITLE-ABS-KEY ( knowledge OR attitudes OR perception OR utilisation ) AND TITLE-ABS-KEY ( afghanistan OR bahrain OR djibouti OR egypt OR iran OR iraq OR jordan OR kuwait OR lebanon OR libya OR morocco OR palestine OR oman OR qatar OR pakistan OR "saudi arabia" OR somalia OR sudan OR syrian OR tunisia OR "united arab emirates" OR yemen ) |
| **Cochrane Central Register of Controlled Trials** | (perimenopause OR menopause):ti,ab,kw AND ("hormone therapy" OR hrt):ti,ab,kw AND (knowledge OR attitudes OR perception OR utilisation):ti,ab,kw AND (afghanistan OR bahrain OR djibouti OR egypt OR iran OR iraq OR jordan OR kuwait OR lebanon OR libya OR morocco OR palestine OR oman OR qatar OR pakistan OR "saudi arabia" OR somalia OR sudan OR syrian OR tunisia OR "united arab emirates" OR yemen):ti,ab,kw |
| **WHO Global Index Medicus** | (perimenopause OR menopause) AND ("hormone therapy" OR "hormone replacement therapy" OR HRT) AND (knowledge OR attitudes OR perception OR utilisation) AND (afghanistan OR bahrain OR djibouti OR egypt OR iran OR iraq OR jordan OR kuwait OR lebanon OR libya OR morocco OR palestine OR oman OR qatar OR pakistan OR "saudi arabia" OR somalia OR sudan OR syria OR tunisia OR "united arab emirates" OR yemen) |
| **Web of Science** | TS=(perimenopause OR menopause) AND TS=("hormone therapy" OR "hormone replacement therapy" OR HRT) AND TS=(knowledge OR attitudes OR perception OR utilisation) AND TS=(Afghanistan OR Bahrain OR Djibouti OR Egypt OR Iran OR Iraq OR Jordan OR Kuwait OR Lebanon OR Libya OR Morocco OR Palestine OR Oman OR Qatar OR Pakistan OR "Saudi Arabia" OR Somalia OR Sudan OR Syria OR Tunisia OR "United Arab Emirates" OR Yemen) |
