# Supplementary material for: Utilisation of hormone replacement therapy in Arab countries: a systematic review
Source: Front Glob Womens Health. 2026 Feb 9;7:1722268. doi: 10.3389/fgwh.2026.1722268 (PMC12926417; doi:10.3389/fgwh.2026.1722268)
Supplement: Supplementary file 3 [file Datasheet3.docx]

Supplementary Table 3: NOS Quality Assessment Done on the 15 Included Studies.

| Author and Year | Selection Bias Assessment (Maximum 5 stars) | | | | Comparability (Maximum 2 stars) | Outcome (Maximum 3 stars) | | Total score (Maximum 10 stars) |
| --- | --- | --- | --- | --- | --- | --- | --- | --- |
|  | Representativeness of the sample | Sample size | Non-respondents | Ascertainment of the exposure (risk factor) | Confounding factors are controlled | Assessment of the outcome | Statistical Test |  |
|  | score | score | score | score | score | score | score |  |
| **Shahzad et al. (2021)** | 0 | 2 | 1 | 1 | 1 | 1 | 1 | 7/10 (Moderate) |
| **Hamid et al. (2014)** | 1 | 1 | 1 | 1 | 0 | 1 | 1 | 6/10 (Moderate) |
| **Ibrahaim & Hussein (2016)** | 1 | 2 | 1 | 1 | 1 | 1 | 1 | 8/10 (High) |
| **Jassim & Al-Shboul (2009)** | 1 | 1 | 1 | 1 | 1 | 0 | 1 | 6/10 (Moderate) |
| **Loutfy et al. (2006)** | 1 | 1 | 1 | 1 | 1 | 1 | 1 | 7/10 (Moderate) |
| **Smail et al. (2020)** | 1 | 1 | 1 | 1 | 0 | 1 | 1 | 6/10 (Moderate) |
| **Mustafa & Sabir (2012)** | 1 | 2 | 1 | 1 | 0 | 1 | 1 | 7/10 (Moderate) |
| **Salem et al. (2020)** | 1 | 1 | 1 | 1 | 0 | 2 | 1 | 7/10 (Moderate) |
| **Albaqami et al. (2023)** | 0 | 2 | 1 | 1 | 0 | 1 | 1 | 5/10 (Low) |
| **Algrnan et al. (2020)** | 0 | 2 | 1 | 1 | 1 | 1 | 1 | 6/10 (Moderate) |
| **Alswayed et al. (2024)** | 1 | 2 | 1 | 1 | 1 | 1 | 1 | 8/10 (High) |
| **Bakarman et al. (2003)** | 1 | 2 | 1 | 1 | 1 | 1 | 1 | 7/10 (Moderate) |
| **Albdour et al. (2021)** | 0 | 1 | 1 | 1 | 0 | 2 | 1 | 6/10 (Moderate) |
| **Tossoun et al. (2014)** | 0 | 1 | 1 | 1 | 0 | 1 | 1 | 5/10 (Low) |
| **Albeitawi et al. (2024)** | 1 | 1 | 1 | 1 | 1 | 1 | 1 | 7/10 (Moderate) |
